# GABAergic Modulation and Neurobehavioral Effects of Arjunolic Acid, a Pentacyclic Triterpene Isolated From *Combretum Mellifluum* Eichler: In Vivo and Molecular Docking Evaluation

**DOI:** 10.1002/cbdv.202502547

**Published:** 2026-03-14

**Authors:** Yanna Julie da Silva Freitas, Jéssica Bezerra Maciel, Maria Eduarda Uchoa Bezerra, Victor Moreira de Oliveira, Simone Alves Serafim Rocha, Otília Loiola Pessoa, Marcia Machado Marinho, Emmanuel Silva Marinho, Cléia Rocha de Sousa Feitosa, Jane Eire Silva Alencar de Menezes, Andreia Ferreira de Castro Gomes, Hélcio Silva dos Santos

**Affiliations:** ^1^ Graduate Program in Natural Sciences State University of Ceará Fortaleza Ceará Brazil; ^2^ Department of Organic and Inorganic Chemistry Federal University of Ceará Fortaleza Ceará Brazil; ^3^ Center For Molecular and Environmental Biology School of Sciences Department of Biology University of Minho Braga Portugal; ^4^ Center For Exact Sciences and Technology State University of Vale do Acaraú Sobral Ceará Brazil

**Keywords:** anxiolytic, anticonvulsant, terpene, GABA receptor, triterpene

## Abstract

This study investigated the neuropharmacological effects of arjunolic acid, with a particular focus on its anxiolytic and anticonvulsant properties. To this end, in vivo tests were performed on adult zebrafish (Danio rerio) and in silico molecular docking analyses were conducted. Acute toxicity (96 h) was evaluated with doses of 4, 20, and 40 mg/kg (*i.p*.). Motor activity and anxiety levels induced by the different doses were evaluated using open field and light/dark tests, while the anticonvulsant potential was tested by induction with pentylenetetrazol (PTZ). The GABAergic neuromodulation was also investigated using the antagonist flumazenil and the molecular interaction with the GABA_A_ receptor and carbonic anhydrase II (CAII). The results demonstrated that arjunolic acid is not toxic at the tested doses, but it does cause significant motor alterations, like those caused by diazepam. The compound exhibited an anxiolytic effect and increased the latency to the onset of convulsive seizures. These effects were reversed by flumazenil, confirming mediation by the GABAergic system. These results corroborate the in silico study, which demonstrated a possible allosteric effect of arjunolic acid on the diazepam binding region of the GABA_A_ receptor and on the active site of CAII. However, arjunolic acid is pharmacologically relevant to the central nervous system and may serve as a basis for the development of new therapeutic agents.

## Graphical abstract

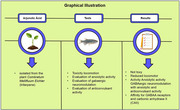

## 1 Introduction

Anxiety is a natural physiological response to dangerous situations; however, when worry and fear become excessive, they constitute Generalized Anxiety Disorder (GAD). This disorder is characterized by symptoms such as abdominal discomfort, tremors, dizziness, palpitations, psychomotor agitation, and tachycardia [1]. The literature shows that anxiety is present in approximately 10% to 30% of epileptic patients, and both conditions may share similar neurobiological mechanisms [2]. Com destaque para a desregulação do sistema do ácido gama‐aminobutírico (GABA). The GABA_A_ receptor is the main target for controlling neuronal excitability; its positive modulation results in immediate anxiolytic and anticonvulsant effects, making it the central focus in the investigation of new compounds that seek to restore neurochemical balance in these comorbidities [3, 4].

Despite the efficacy of current benzodiazepines and anticonvulsants, clinical management is limited by adverse effects such as severe sedation, tolerance, and dependence [5, 6]. In this scenario, triterpenes such as arjunolic acid, isolated from *Combretum mellifluum* Eichler (a plant popularly known as cipó, cipó‐vermelho, mofumbo, or cipaúba, native to the Brazilian Cerrado biome), emerge as promising candidates. The choice of this compound is based on its lipophilic structure, which facilitates permeability in biological membranes, and on evidence that analogous triterpenes act as allosteric modulators of GABA_A_ receptors, offering a safety profile potentially superior to synthetic drugs [7, 8]. In addition, arjunolic acid has been reported as a promising compound because it has antioxidant, antitumor, antifungal, anticholinergic, antibacterial, and healing actions [9, 10].

In the search for effective therapeutic alternatives, animal models such as zebrafish (*Danio rerio*) have been widely used due to their applicability in behavioral and molecular studies focused on neurological disorders [11, 12]. By expressing all GABAergic receptors and encoding proteins associated with the GABA receptor, this animal model is essential for neurochemical study, screening, and development of drug therapies for diseases caused by changes in GABAergic neurotransmission, such as anxiety and seizures [13]. This model also has a functional and permeable blood‐brain barrier, which favors the analysis of compounds with action on the central nervous system. [14] At the same time, the use of in silico approaches, such as molecular docking, has proven to be an effective tool in identifying interactions between ligands and their biological targets, contributing significantly to the process of discovering new drugs [15].

In light of the above, through exploratory research, this study aimed to evaluate the toxic, sedative, anxiolytic, and anticonvulsant effects of arjunolic acid, isolated from *C. mellifluum* Eichler, in adult zebrafish, as well as to investigate its molecular interactions through molecular docking, with the aim of contributing to the development of new therapeutic candidates against the comorbidities of anxiety and seizures.

## 2 Results and Discussion

### 2.1 Acute Toxicity 96 h

The zebrafish animal model has been applied in toxicological testing of different pollutants [16, 17] and is also used in the toxicity assessment of pharmaceutical products [18, 19, 20]. With this, zebrafish was used to evaluate the toxic effect of arjunolic acid, which was shown not to be toxic to adult zebrafish up to 96 h of analysis (LD_50_ > 40 mg/kg) as shown in (Table 1), as there was no significant number of deaths and no apparent anatomical changes in the animals during this period (p > 0.05).

**TABLE 1:** Results of acute toxicity tests for Arjunolic Acid.

Sample	Mortality				96 h LD_50_ (mg/kg) / IC
	CN	D1	D2	D3	
**Arjunolic acid**	0	0	0	0	> 40

Legend: CN—Negative control group: DMSO 3%. D1 ‐ Dose 1 (4 mg/kg). D2 ‐ Dose 2 (20 mg/kg). D3 ‐ Dose 3 (40 mg/kg). LD50 ‐ lethal dose to kill 50% of adult zebrafish; CI—confidence interval.

### 2.2 Locomotor Activity (Open Field Test)

**FIGURE 1:**
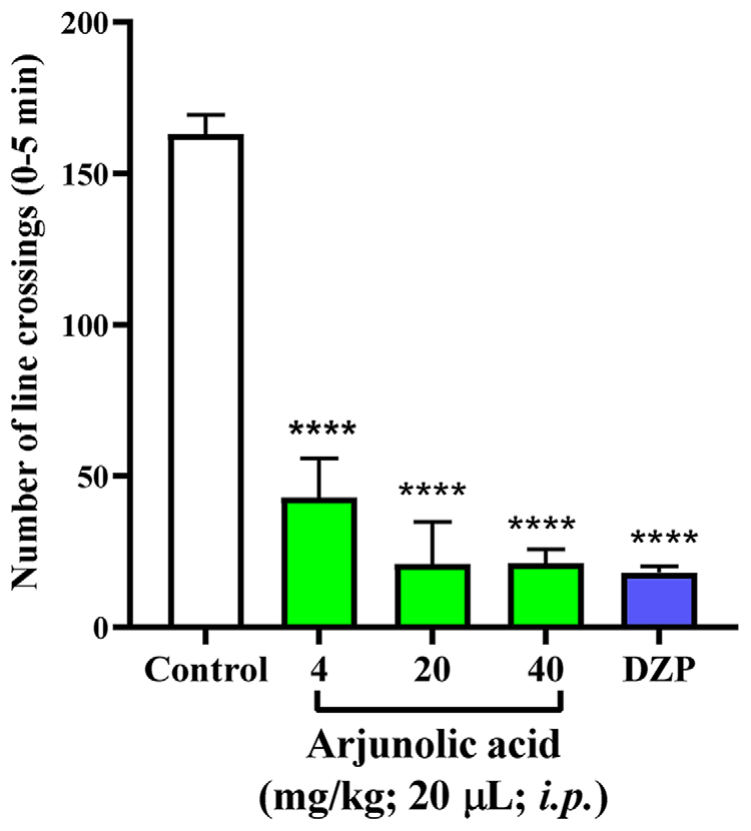
Effect of arjunolic acid on locomotor behavior of adult zebrafish in the Open Field Test (0–5 min). Values represent the mean ± standard error of the mean for 6 animals/group; ANOVA followed by Tukey's test. (****p<0.0001; vs. Control).

Locomotor activity is a parameter considered in order to evaluate, through behavior, the effect of chemical substances on the central nervous system (CNS) of the zebrafish [20]. Thus, locomotor activity can be analyzed using the open field test, which evaluates different parameters such as freezing (immobility) [3]. According to the one‐way analysis of variance (ANOVA), arjunolic acid at doses of (4, 20, and 40 mg/kg) showed different behavior compared to the negative control (DMSO3%) (****p<0.0001 vs. DMSO3%), demonstrating a change in the animal's locomotion (Figure 1). With this, all doses showed similar behavior to the negative control Diazepam (DZP), with the animals demonstrating a more lethargic behavior. The results of arjunolic acid were similar to those obtained with anxiolytic drugs that caused a sedative effect and decreased locomotor activity in animals [21, 22]. According to the study by Johnson [23], other types of terpenes showed a change in the locomotor activity of zebrafish, because terpenes interact with the GABA_A_ receptor complex to prolong GABAergic synaptic transmission, favoring its potential sedative and anxiolytic effects.

### 2.3 Evaluation of Anxiolytic Activity

**FIGURE 2:**
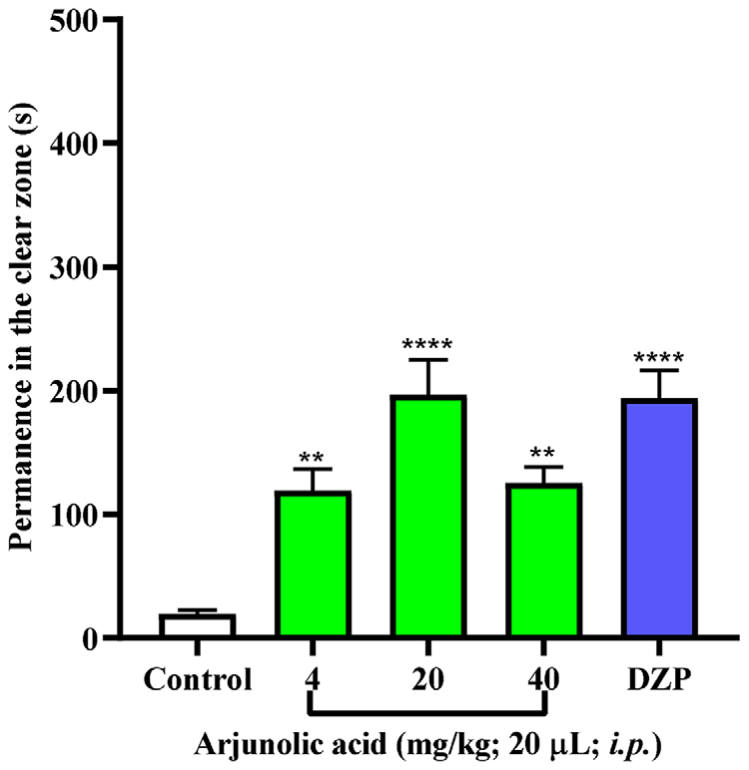
Arjunolic acid light/dark test. Anxiolytic effect of samples in adult zebrafish (Danio rerio) by the Light/Dark Test (0‐5 min). Control (DMSO 3%; 20 µL; i.p.); Dzp—Diazepam (4 mg/kg; i.p.). Values represent the mean ± standard error of the mean (S.E.M.) for 6 animals/group; ANOVA followed by Tukey. (**p<0.01, ****p<0.0001 vs. Control).

Anxiety medications act on specific neurotransmitter systems such as norepinephrine (NE), 5‐hydroxytryptamine (5‐HT, serotonin), and gamma‐aminobutyric acid (GABA) [24]. Triterpenes exhibit both serotonergic and GABAergic neuromodulation, possessing anxiolytic and anticonvulsant activity in mice and adult zebrafish [25, 26]. To investigate anxiety‐modulating drugs and their mechanism of action, the light‐dark test in zebrafish is an essential tool. [27] With this, arjunolic acid in adult zebrafish showed that at all doses (4, 20, and 40 mg/Kg), there was a significant difference from the negative control (DMSO 3%), indicating a possible reversal of the animal's natural anxious behavior (Figure 2). Furthermore, all concentrations, especially the concentration of 20 mg/kg, revealed results similar to the behavior presented by the positive control Diazepam, evidencing a potential anxiolytic effect, with animals spending more time in the light zone of the aquarium.

### 2.4 Evaluation of GABAergic Neuromodulation

**FIGURE 3:**
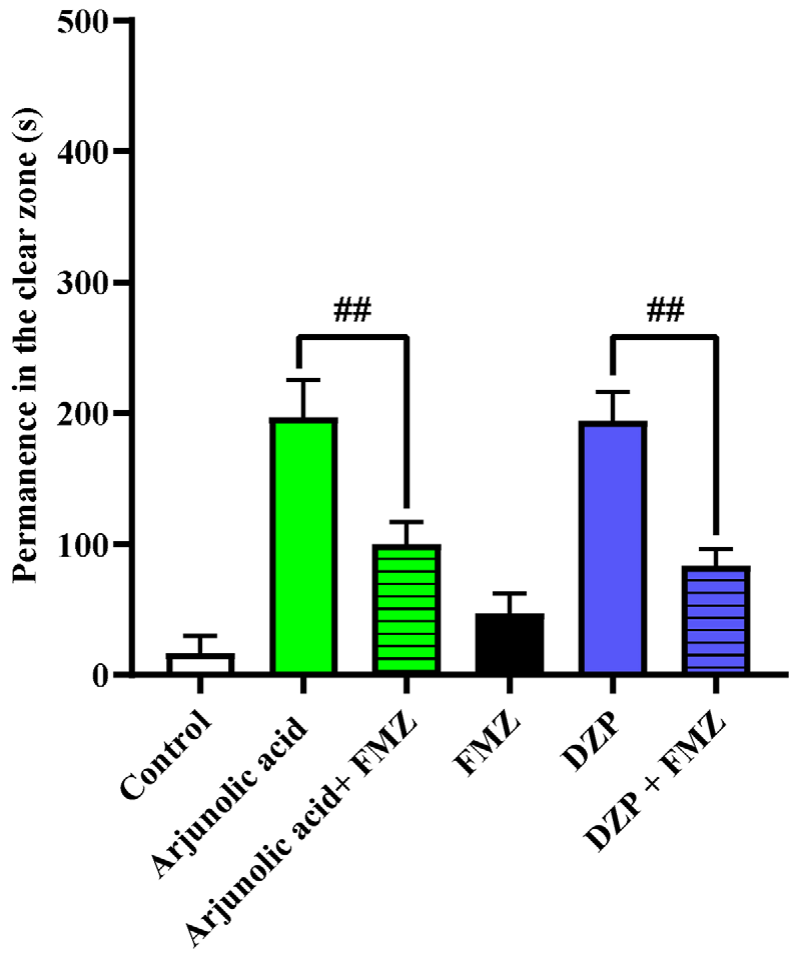
Mechanism of anxiolytic action via GABA of arjunolic acid (20 mg/kg) in adult (Danio rerio) in the Light & Dark Test (0‐5 min). Control—DMSO 3% (20 µL; i.p.). DZP—Diazepam (4 mg/kg; 20 µL; i.p.). Values represent the mean ± standard error of the mean (S.E.M.) for 6 animals/group. ANOVA followed by Tukey (##p<0.01 vs. Fmz + CM 5; ##p<0.01 vs. Fmz + DZP).

Gamma‐aminobutyric acid is the main inhibitory neurotransmitter of the central nervous system (CNS). GABA exerts its effect through three types of receptors, grouped as GABA_A_, GABA_B_, and GABA_C_. BZDs bind to GABA_A_ receptors, causing positive modulation of the receptor and increasing GABA activity; receptors containing α 2 βγ 2 and α 3 βγ 2 subunits are responsible for promoting the anxiolytic and muscle relaxant effects of these drugs [25]. Flumazenil is a benzodiazepine antagonist used in cases of overdose or in the reversal of the sedative effects of benzodiazepines associated with anesthesia [28]. The antagonist has a high‐affinity binding site on the extracellular surface of the GABA_A_ receptor at an interface between the α and γ 2 subunits, close to the benzodiazepine binding site. In this way, flumazenil occupies the benzodiazepine receptor, blocking its action [29, 30]. Based on this principle, flumazenil can be used to track the mechanism of action of drugs that act via the GABA pathway [27]. The anxiolytic effect of arjunolic acid (20 mg/kg) was neuromodulated by the GABA_A_ receptor, as it showed a significant statistical difference (arjunolic acid ##p<0.01 vs. arjunolic acid+Fmz), as did DZP, which obtained a significant difference with DZP+FMZ, showing an anxiolytic effect (##p<0.01 vs. Fmz + arjunolic acid; ##p<0.01 vs. Fmz +DZP). Therefore, this means that the anxiolytic effect of arjunolic acid was blocked by flumazenil (Figure 3).

### 2.5 Anticonvulsant Activity

**FIGURE 4:**
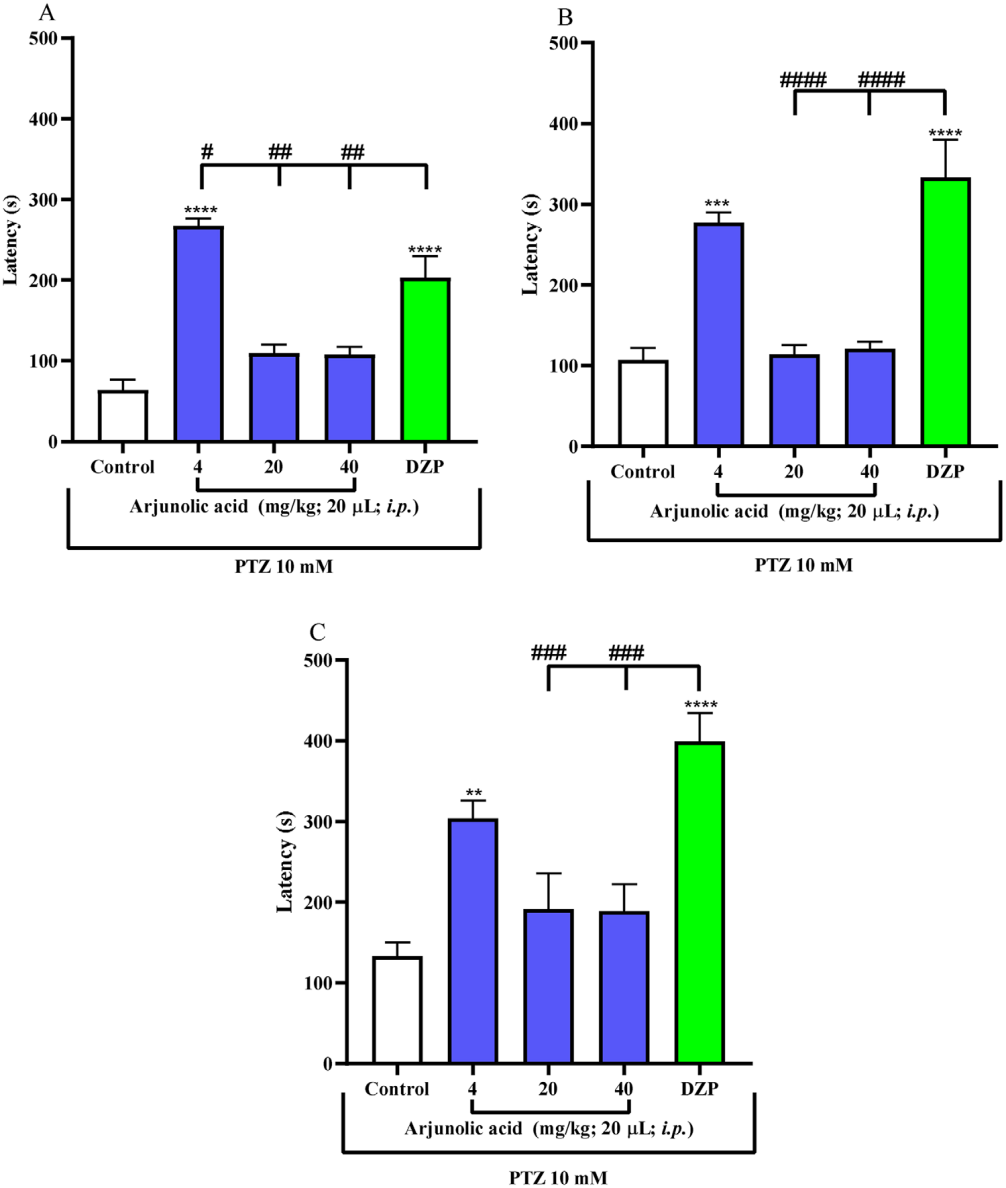
Effect of arjunolic acid on PTZ‐pentylenetetrazol‐induced seizures (A) stage I, (B) stage II, and (C) stage III in adult (Danio rerio). Values represent the mean ± standard error of the mean for six animals/group; ANOVA followed by Tukey's test (**p<0.01; ***p<0.001; ****p < 0.0001 vs. vehicle‐DMSO (3% dimethyl sulfoxide); #p < 0.1; ##p < 0.01; ###p < 0.001; vs. DZP‐Diazepam). DMSO (3% dimethyl sulfoxide).

The results demonstrate that in seizures induced by pethylenetetrazole (Figure 4), arjunolic acid significantly delayed (****p < 0.0001) the three stages of the animal's seizure at a dose of 4 mg/kg. Therefore, animals under the effect of arjunolic acid exhibited behavior similar to that of the reference drug, diazepam (****p < 0.0001).

**FIGURE 5:**
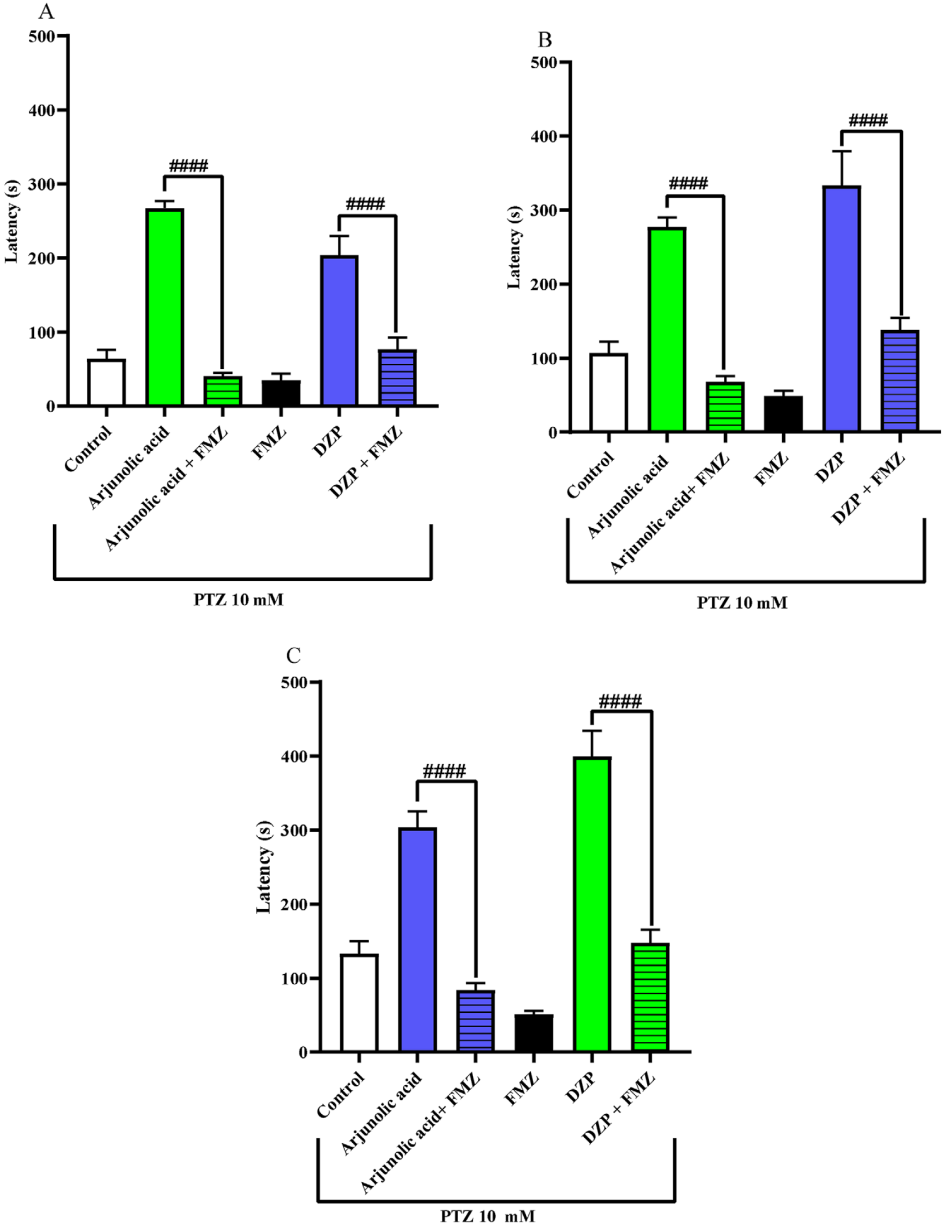
Effect of flumazenil (FMZ) on the anticonvulsant effect of arjunolic acid (4 mg/kg) in the PTZ‐pentylenetetrazol‐induced seizure test in adult (Danio rerio). Values ​​represent the mean ± standard error of the mean for six animals/group; ANOVA followed by Tukey's test; (A) stage I; (B) stage II, and (C) stage III (###p < 0.0001 vs. Arjunolic acid or DZP‐Diazepam). DMSO (3% dimethyl sulfoxide).

Pentylenetetrazol is a convulsant that acts allosterically on the GABA receptor, making it possible to investigate anticonvulsant activity through GABAergic neuromodulation of arjunolic acid. [27] Terpenes are a class of secondary metabolites that have action on the Central Nervous System (CNS), including anxiolytic and anticonvulsant activity [31]. Studies with rats [32] and zebrafish [33] show that terpenic substances or their derivatives have been able to reduce convulsions induced by PTZ, and the anticonvulsant effects and the mechanism may be particularly related to the positive regulation of the levels of the inhibitory neurotransmitter GABA [34, 35]. In addition, Passos et al. [31] present different classes of isolated terpenes that have action mainly on GABAergic neurotransmitter systems [35]. Arjunolic acid, because it is included in this class, also showed GABAergic neuromodulation and, because of this, showed in the results a delay of the three stages of convulsions submitted with PTZ in zebrafish (Figure 5). Therefore, arjunolic acid presented both anxiolytic and anticonvulsant effects, and it is quite common for the benzodiazepine class to present both activities for the treatment of diseases [36]. Our findings suggest that the lowest dose (4 mg/kg) may represent the optimal therapeutic window for arjunolic acid in this model, as higher doses may lead to receptor desensitization, binding site saturation, or the recruitment of counterproductive pathways (such as effects on excitatory systems) that may partially mask the anticonvulsant effect [37]. Figure 5

### 2.6 Molecular Docking

#### 2.6.1 GABA_A_ Receptor (GABA_A_R)

**FIGURE 6:**
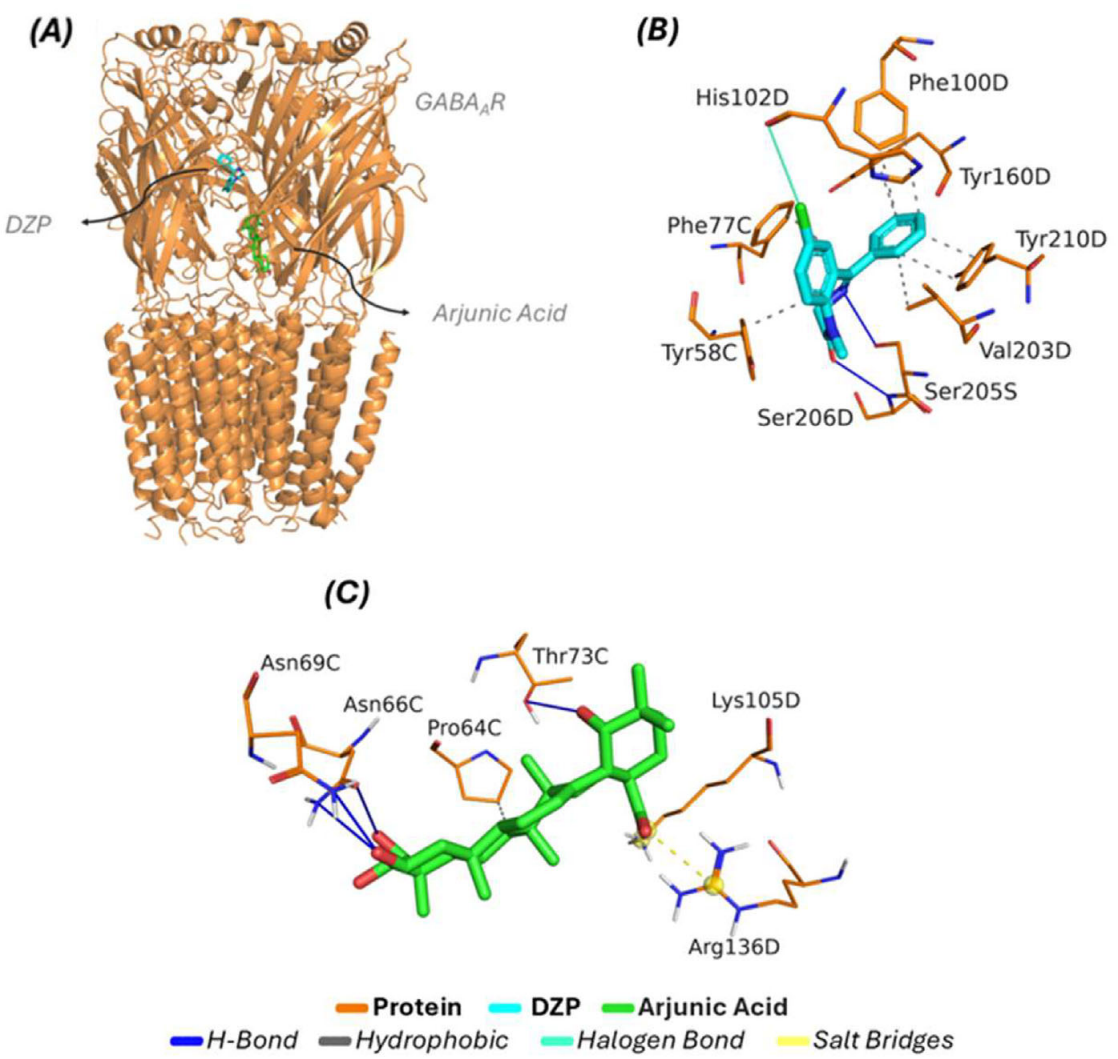
Molecular docking simulations for the GABA_A_R receptor (A) global projection of the complexes formed, (B) interactions formed by the presence of the inhibitor DZP, (C) formation of interactions by the ligand arjunic acid.

To elucidate the potential mechanism of action acting upon the GABA_A_R, Figure 6 (A) presents an overview of the complexes formed through the relationship between ligand and protein. According to the results expressed through molecular docking analysis, the respective interaction energy values and RMSD variations associated with the DZP/GABA_A_R complex were observed (−7.2 kcal/mol and 1.967 Å), respectively; while for the arjunolic acid/GABA_A_R complex, an RMSD variation of 1.956 Å and an energy value equivalent to −7.9 kcal/mol were indicated. With the proper formation of a complex between ligand and protein, Figure 6 (B) detects all the interactions formed between the crystallized ligand DZP (inhibitor), associated by affinity with the interaction cavity, highlighting eight contributions with hydrophobic characteristics through the presence of the amino acid residues TYR58C (3.50 Å), PHE77C (3.46 Å), PHE100D (3.84 Å), PHE100D (3.91 Å), TYR160D (3.55 Å), VAL203D (3.96 Å), TYR210D (3.35 Å), and TYR210D (3.88 Å); while regarding the hydrogen bond, two bonds were presented with the residues SER205D and SER206D with distance values equal to (2.95 Å) and (3.17 Å), respectively; as for the interactions linked to the halogenated profile, the contribution of the amino acid HIS102D is highlighted; the other data associated with the complex formed is available in Table 2. Molecular docking prediction studies aim to elucidate the mechanism of action resulting from complex formation with the presence of the arjunolic acid ligand through the interactions formed between the compound and the amino acid residues present in the interaction cavity. In light of the studies, a hydrophobic interaction with the residue PRO64C (3.97 Å) is indicated, while for hydrogen bonds, the formation of four bonds with the amino acid residues ASN66C (3.19 Å), ASN66C (3.46 Å), ASN69C (2.59 Å), and THR73C (2.76 Å) is perceptible; and finally, two interactions referring to the salt bridges style with two amino acid residues (LYS105D and ARG136D), presenting the shortest distance equal to 3.35 regarding the contribution of the residue ARG136D. The data from the molecular docking simulations show that the compound arjunolic acid presented a different interaction profile when compared to the crystallized ligand DZP (inhibitor). These results suggest that the possible biological activity linked to the compound arjunolic acid is apparently associated with non‐competition for the inhibition site cavity, thus aiming at a possible allosteric effect.

**TABLE 2:** Distance values linked to complexes formed with the GABA_A_R receptor.

Complex	Energy (kcal/mol)	Interaction Type	Residue (Distance in Å)
DZP/GABA_A_R	−7.2	Hydrophobic	TYR58C (3.50), PHE77C (3.46), PHE100D (3.84), PHE100D (3.91), TYR160D (3.55), VAL203D (3.96), TYR210D (3.35), and TYR210D (3.88)
		H‐Bond	SER205D (2.95) and SER206D (3.17)
		Halogen Bond	HIS102D (3.80)
Arjunolic acid/GABA_A_R	−7.9	Hydrophobic	PRO64C (3.97 Å)
		H‐Bond	ASN66C (3.19), ASN66C (3.46), ASN69C (2.59) and THR73C (2.76)
		Salt Bridges	LYS105D (4.98) and ARG136D (3.35)

Legend: DZP/GABA_A_R: Diazepam bound to its active site on the GABA_A_ receptor; Arjunolic acid/GABA_A_R: Arjunolic acid binding site on the GABA_A_ receptor.

#### 2.6.2 Carbonic Anhydrase II (CAII) and Its Active Site for Anticonvulsant Activity

**FIGURE 7:**
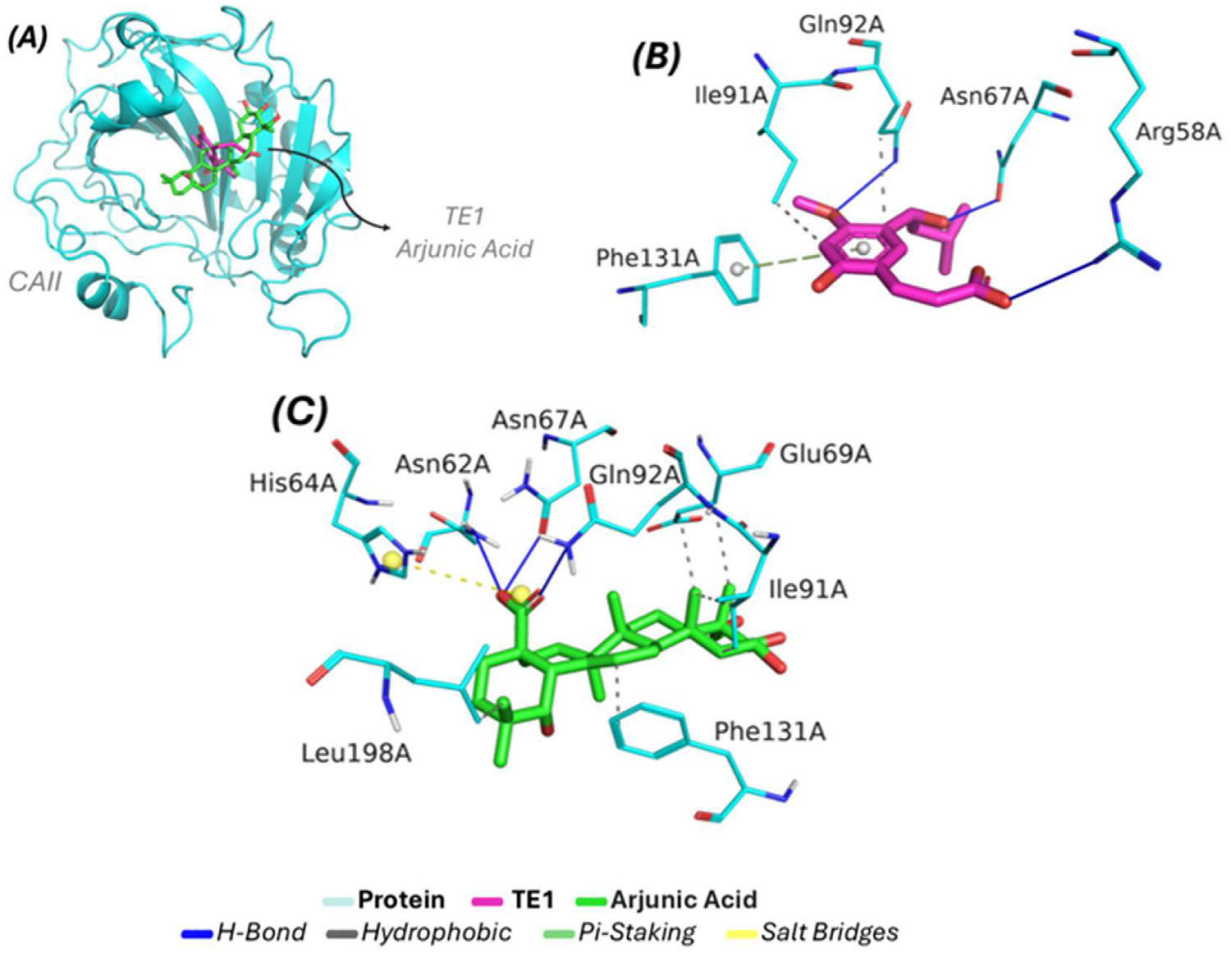
Molecular docking simulations against the CAII receptor. (A) global affinity of both complexes (B) interactions formed by the contribution of the inhibitor (C) Projection of the firm interactions with the arjunic acid ligand.

Molecular docking studies aim to apply the principle of affinity resulting from the relationship between the ligand arjunic acid and the receptor CAII. According to the simulation data, Figure 7 (A) shows the affinity profile resulting from the relationship between the ligand and protein, highlighting that the inhibitor TE1 (pink) forms a complex with the protein, with energy and RMSD values of −5.6 kcal/mol and 1.965 Å, respectively; while the compound arjunic acid (green) expressed its complex form with values equal to −6.7 kcal/mol (affinity energy) and 1.751 Å (RMSD). Based on the analysis of interactions, Figure 7 (B) shows all the interactions formed between the ligand TE1 and the amino acids inserted in the catalytic cavity. The studies indicate two hydrophobic interactions contributed by the amino acid residues ILE91A (3.65 Å) and GLN92A (3.55 Å); the inhibitor presented three hydrogen bonds formed by three distinct amino acid residues (ARG58A, ASN67A, and GLN92A), with the shortest distance associated with the residue ASN67A (1.42Å), and finally a pi‐stacking interaction with the contribution of PHE131A (4.77 Å). These residues are relevant to the inhibition profile linked to the CAII receptor. When evaluating the behavior of the interactions formed by the presence of arjunic acid, Figure 6 (C) shows all the interactions established with the application of the ligand in the interaction cavity. Among the projections, six hydrophobic interactions were observed with four amino acid residues (GLU69A, ILE91A, PHE131A, and LEU198A), providing the shortest distance equal to 3.11 Å (ILE91A); while for the hydrogen bonds, three contributions from amino acid residues were found (ASN62A, ASN67A, and GLN92A) presenting distance values equal to (2.60 Å), (2.05 Å), and (2.22 Å), respectively; furthermore, the presence of the ligand arjunic acid played a role in the formation of a salt bridge interaction with the amino acid HIS64A (5.45 Å). All distance values linked to the types of interactions and their complexes are included in Table 3. The data resulting from the molecular docking simulations indicated that the ligand of interest, arjunic acid, expressed affinity in the same region as the crystallized inhibitor, directly corroborated by the predominance of four amino acid residues (GLN67A, ILE91A, GLN92A, and PHE131A), since both ligands share favorable interactions. These results show that the arjunic acid ligand presents an inhibition profile linked to the competitive effect on the catalytic site when compared to the crystallized inhibitor.

**TABLE 3:** Distance values for complexes formed with the CAII receptor.

Complex	Energy (kcal/mol)	Interaction Type	Residue (Distance in Å)
TE1/CAII	−5.6	Hydrophobic	ILE91A (3.65) and GLN92A (3.55)
		H‐Bond	ARG58A (3.34), ASN67A (1.42) and GLN92A (2.33)
		Pi‐Staking	PHE131A (4.77)
Arjunic Acid/CAII	−6.7	Hydrophobic	GLU69A (3.91), GLU69A (3.71), ILE91A (3.68), ILE91A (3.11), PHE131A (3.93) and LEU198A (3.71)
		H‐Bond	ASN62A (2.60), ASN67A (2.05) and GLN92A (2.22)
		Salt Bridges	HIS64A (5.45)

Legend: TE1/CAII: cocrystallized inhibitor bound to the active site of carbonic anhydrase II; Arjunic Acid/CAII: Arjunic acid binding site in carbonic anhydrase II enzyme.

Previous studies conducted with the ethanolic extract of Combretum lanceolatum, which exhibited anxiolytic, sedative, and anticonvulsant effects, revealed, through in silico analysis, the interaction of glycosylated flavonoids at the active site of the CAII enzyme. These compounds interacted via hydrophobic contacts with residues GLU69, ILE91, and GLN92, in addition to establishing hydrogen bonds with ASN62, GLN92, and ARG58 at the catalytic site, and with adjacent residues HIS64, ASN67, ASP72, THR200, and PRO201 [38]. These data corroborate the findings obtained for the triterpene arjunolic acid, which showed a similar interaction profile with the same amino acid residues at the active site of CAII. This molecular convergence explains why arjunolic acid is so good at slowing down seizures in zebrafish models, even at the lowest dose tested, suggesting that modulating CAII is key to its anticonvulsant activity.

### 2.7 Molecular Dynamics Simulations

**FIGURE 8:**
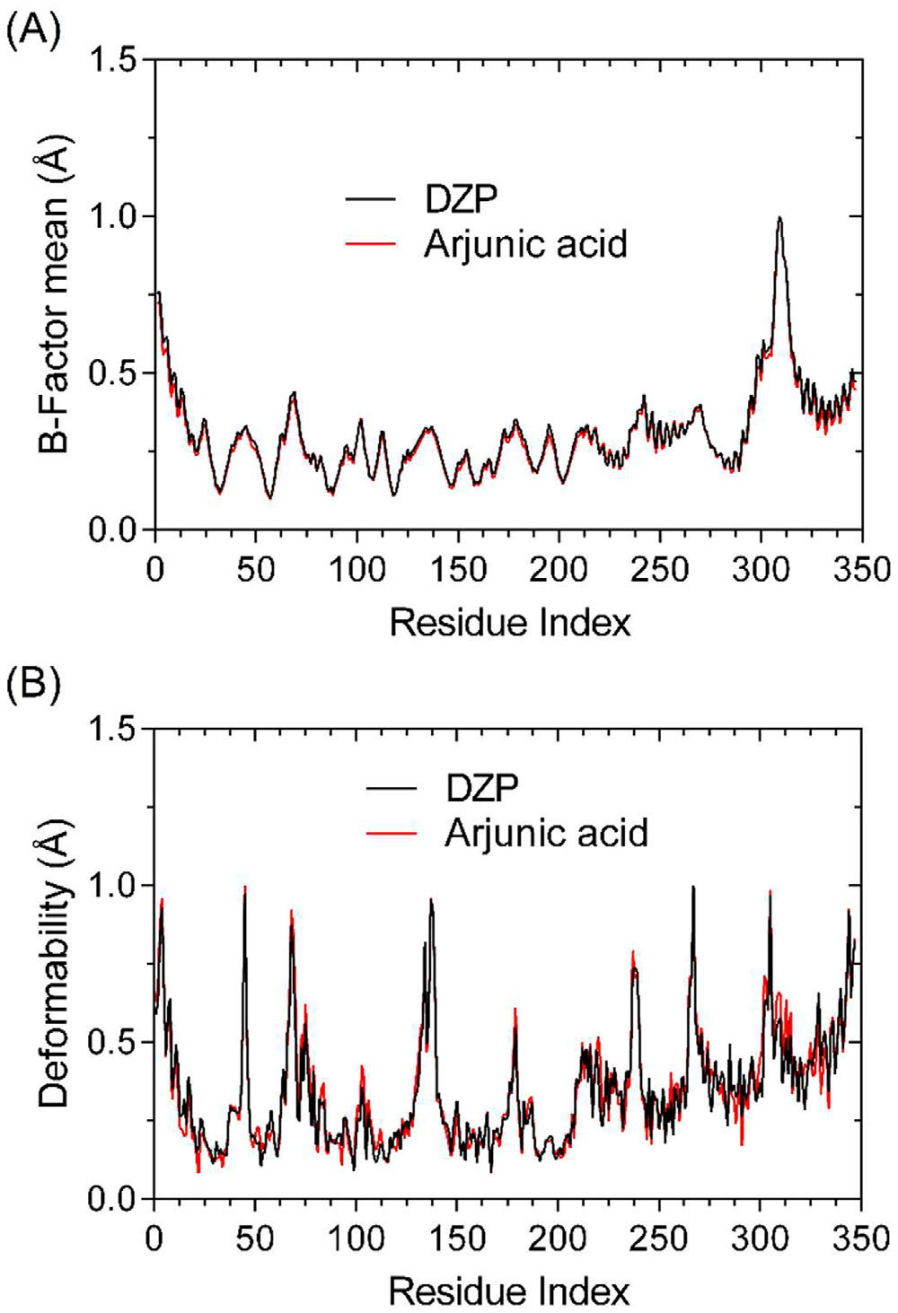
Dynamics simulations with the GABA A receptor (A) highlight conformational variations (B) Deformability profile.

Conformational studies and preliminary analyses based on Normal Mode Analysis (NMA) have revealed that the system constructed with the protein‐ligand complex exhibits overall structural stability, with conformational variations being restricted to specific regions of the protein. As demonstrated in Figure 8(A), variations related to Cα atoms associated with the GABA A receptor manifest in a localized manner, devoid of the predominance of abrupt variations between systems. This observation is consistent with harmonic fluctuations anticipated for stable protein systems, thereby suggesting the absence of global structural disturbances induced by ligand binding.

Furthermore, Figure 8(B) demonstrates that the regions exhibiting increased flexibility are predominantly associated with segments of the structure that are naturally more mobile, such as loop regions, without compromising the structural integrity of the GABA A receptor core. This behaviour suggests that ligand accommodation promotes only local conformational adjustments, thereby preserving the integrity of protein folding.

Overall, the combined information in Figure 7 suggests that the complex formed is structurally viable and stable, providing a consistent initial basis for subsequent comparative analyses of deformability and relative stability compared to other reference ligands.

Normal mode analysis (NMA) calculated for the enzyme carbonic anhydrase II (CA II) indicates that both the complex formed with the TE1 compound and that formed with the proposed arjunic acid maintain overall structural integrity. As illustrated in Figure 8A, the fluctuations observed in the Cα atoms along the protein backbone remain within a range characteristic of stable systems due to low structural variations.

**FIGURE 9:**
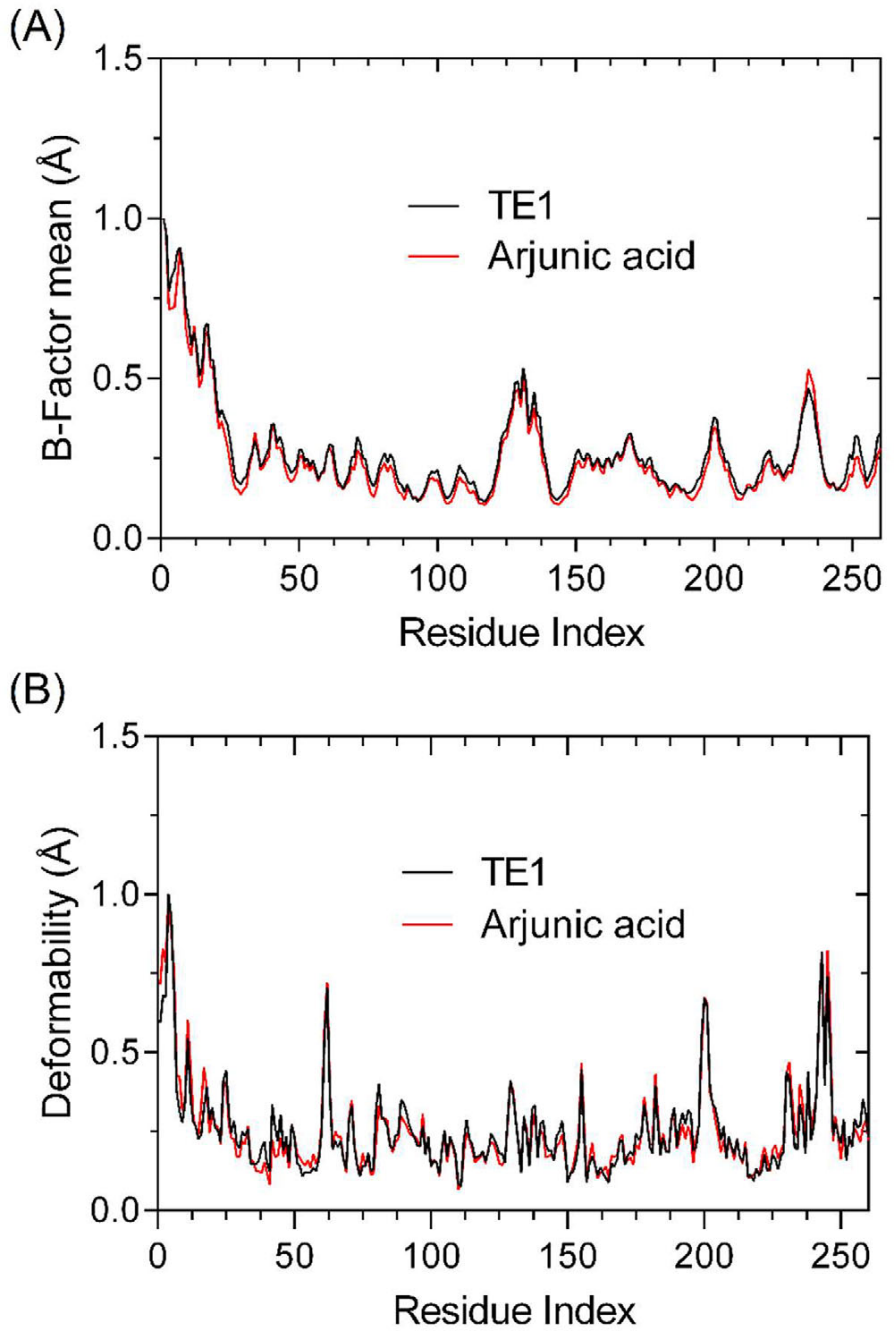
Dynamics simulations with the CAII receptor (A) highlight conformational variations (B) Deformability profile.

As illustrated in Figure 8B, the segments exhibiting greater flexibility are localised in specific portions of particular loops of the respective biological receptor, which are intrinsic to the mobility of the respective protein. The central catalytic site, in turn, exhibits preserved conformational rigidity in both complexes. A direct comparison between the dynamic profiles of TE1 and arjunic acid reveals that the proposed ligand promotes only localized and restricted conformational readjustments, exhibiting a structural fluctuation pattern similar to and compatible with that of the established reference inhibitor (Figure 9).

**FIGURE 10:**
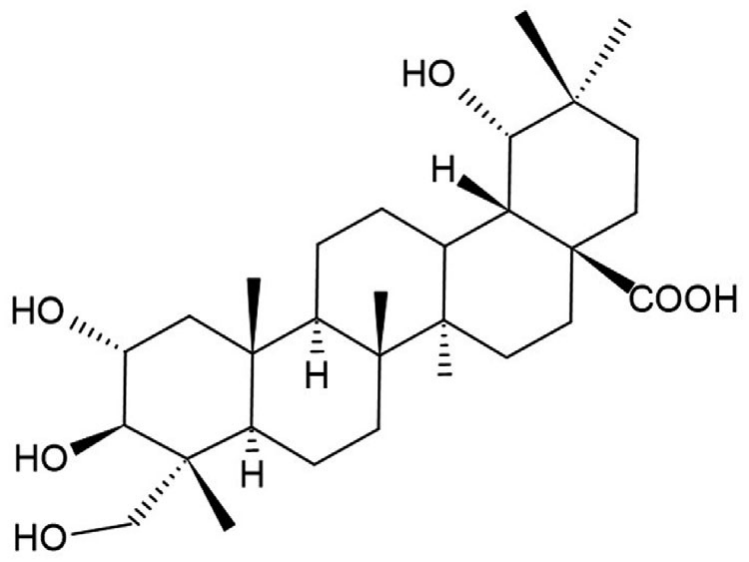
Chemical Structure of Arjunolic Acid.

As shown in Figures 7 and 8, the data demonstrate that both systems constructed with GABA A and CAII receptors exhibit a high degree of conformational stability, linked by low conformational variations caused by the formation of a complex between the ligand and the protein. The data presented here indicate that the complexes under analysis exhibit high affinity and specificity. This finding is further supported by molecular docking simulations, which directly corroborate the potentially favorable biological effect of the targets studied (Figure 10).

## 3 Conclusions

In summary, this study provides robust evidence that arjunolic acid exhibits a multifaceted pharmacological profile, acting as a sedative, anxiolytic, and anticonvulsant agent in adult zebrafish models. The added value of this research lies in the identification of a novel phytocompound with low acute toxicity and high neuropharmacological selectivity. The reversal of the anxiolytic effect by flumazenil, together with molecular docking and trajectory analyses, supports the hypothesis that arjunolic acid acts as a modulator of the GABA_A_ receptor complex, while also interacting with carbonic anhydrase II (CAII). In silico studies demonstrated a high binding affinity and conformational stability of the arjunolic acid GABA_A_ and arjunolic acid–CAII complexes, which are favorably associated with anxiolytic and anticonvulsant effects. These findings suggest that arjunolic acid may represent a promising alternative to traditional benzodiazepines.

## 4 Experimental Section

### 4.1 Medications and Reagents

The following substances were used: Diazepam (DZP, Neo Química), Dimethyl sulfoxide (3% DMSO; Dynamic), flumazenil (FMZ, Roche Pharmaceutical), and pentylenetetrazol (PTZ, Sigma‐Aldrich).

### 4.2 Material Acquisition

The plant material was collected in Tianguá, Ceará, Brazil, from a *C. mellifluum* Eichler shrub in the fruiting stage (Coordinates: −3.6298 S, −40.9776 W; Elevation: 705 m). Botanical identification was confirmed, and a voucher specimen was deposited at the Prisco Bezerra Herbarium (UFC) under registration number EAC0049081. The crude leaf extract was obtained according to Braquehais et al. [39], with minor modifications. Leaves were air‐dried (30 ± 2 °C), ground, and subjected to exhaustive cold maceration in 96% commercial ethanol for 96 h. The resulting solution was filtered under aseptic conditions and concentrated using a water bath at 50 °C. The extract was stored at 5 °C until further use. Arjunolic acid (Figure 8) was isolated from the ethanolic extract of the leaves and provided by Prof. Otília Deusdênia Loiola Pessoa, head of the Natural and Marine Products Laboratory (Department of Organic and Inorganic Chemistry, UFC).

### 4.3 Animals

Zebrafish (*Danio rerio*) (90 to 120 days old; 0.4 ± 0.1 g, 3.5 ± 0.5 cm), wild‐type, of both sexes, were acquired from a local store (Fortaleza, CE, Brazil). The animals were kept in a 10 L glass aquarium (30 × 15 × 20 cm) (n = 3 / L), at a temperature of 25 ± 2°C, in 24 h light‐dark cycles with chlorinated water (ProtecPlus) and an air pump with submerged filters, under a temperature of 25°C and pH 7.0, Circadian cycle of 10 ‐ 14 h (light/dark). The fish received feed (Spirulina) ad libitum 24 h before the experiments. Before drug applications, the animals were anesthetized in ice water, and after the experiments, the animals were sacrificed by immersion in ice water (2 and 4°C) for 1 min until loss of opercular movements. The work was approved by the Ethics Committee on Animal Use of the State University of Ceará (CEUA‐UECE; n° 04983945/2021), and is in accordance with the Ethical Principles of Animal Experimentation.

### 4.4 96 h Acute Toxicity

The fish (n = 6 / group) were treated intraperitoneally (*i.p*.) with 20 µL of arjunolic acid samples (4, 20, and 40 mg/kg, 20 µL; i.p) diluted in 3% DMSO. 3% DMSO was used as a negative control. After 24, 48, 72, and 96 h, the values obtained with the number of dead ZFa were submitted to statistical analysis, estimating the lethal dose to kill 50% (LD_50_) Zebrafish (Zfa) using the Trimmed Spearman‐Karber mathematical method with 95% confidence intervals [18]. Non‐toxic doses were selected to investigate the effects on anxiety‐like behavior and seizure activity in zebrafish. The selection of these doses was based on protocols established in the literature [3, 4], which demonstrate that low, non‐toxic doses of compounds maintain the physiological integrity of animals while promoting significant anxiolytic and anticonvulsant effects.

### 4.5 Open Field Test

The experiment was performed according to the methodology of Ahmad and Richardson [40] and with adaptations [19], to evaluate the locomotor activity of adult zebrafish under the action of analgesic drugs [41]. The zebrafish (n = 6/group) were treated intraperitoneally (*i.p*.) with arjunolic acid at doses of (4, 40, and 40 mg/kg), a vehicle group (3% DMSO, 20 µL; *i.p*.) as a negative control, and a DZP group (4 mg/kg; 20 µL; *i.p*.) was used as a positive control. After 30 min, the groups were individually placed in Petri dishes marked with four quadrants, and locomotor activity was assessed by the number of line crossings (LC) for 5 min.

### 4.6 Evaluation of Anxiolytic Activity

The anxiety behavior of an animal can be observed through the light/dark test. Similar to rodents, zebrafish naturally avoid illuminated areas [42]. The experiment was performed in a glass aquarium (30 cm x 15 cm x 20 cm) divided into a light area and a dark area. The aquarium was filled to 3 cm with chlorine‐free tap water, which simulated a new shallow environment different from the conventional aquarium and capable of inducing anxiety behaviors. In the animals (n = 6/group), 20 µL of the arjunolic acid sample was administered *i.p*. at doses of 4, 20, and 40 mg/kg. The negative and positive control groups consisted of 3% DMSO and 4 mg/kg Diazepam solution, respectively. After 30 min, the animals were individually placed in the light zone, and the anxiolytic effect was measured based on the time spent in the light zone of the aquarium within 5 min of observation [22].

### 4.7 Evaluation of GABAergic Neuromodulation

The mechanism of action tested for the possible anxiolytic effect of arjunolic acid was performed by pre‐treatment with flumazenil (neutralizing modulator of positive GABA_A_ modulators) [21]. Zebrafish (n = 6/group) were pre‐treated with flumazenil (4 mg/kg; 20 µL; i.p). After 15 min, the best effective dose of arjunolic acid was administered (20 mg/kg; 20 µL; *i.p*.); 3% DMSO (vehicle; 20 µL; i.p) was used as a negative control. DZP (4 mg/kg, 20 µL; *i.p*.) and fluoxetine (0.05 mg/mL; i.p) were used as GABA_A_ agonists, respectively. After 30 min of treatment, the animals were subjected to the light/dark test.

### 4.8 Pentylenetetrazol (PTZ)‐induced Seizure via the GABAergic System

The reversal of PTZ‐induced seizure was investigated. The fish groups (n = 6/group) were treated with arjunolic acid (4, 20, and 40 mg/kg, 20 µL; *i.p*.), Diazepam (4 mg/kg; 20 µL; *i.p*.), and control (3% DMSO; 20 µL; *i.p*.). After 1 h, the animals were individually exposed by immersion in 7.5 mM PTZ, dissolved in water in a 250 mL beaker, and seizure‐like behavior was evaluated in three stages: stage I—dramatic increase in swimming activity; stage II—whirlpool swimming behavior; stage III—clonic‐type seizures, followed by loss of posture when the animal falls to the side and remains immobile for 1‐3s [43] At the end of the evaluation of the 3 stages of the test, the animals were euthanized in ice water.

### 4.9 Statistical Analysis

Results were expressed as mean ± standard error of the mean for each group of 6 animals. After confirming the normality of distribution and homogeneity of the data, differences between groups were subjected to analysis of variance—one‐way or two‐way ANOVA, followed by Tukey's test. All analyses were performed using GraphPad Prism v. 8.0 software. The level of statistical significance was set at 5% (p<0.05).

### 4.10 Molecular Docking

For the simulations, the 2D modeling of arjunolic acid was first performed using MarvinSketch software version 24.1.0, Chemaxon (https://chemaxon.com/marvin) [44]. After ligand standardization, Avogadro software [45] was used to convert the compound to the third dimension, and then it underwent a structural optimization process using the MMFF94 (Merck Molecular Force Field 94) force field method [45, 46] to obtain the lowest energy state for the respective compound and consequently more stable according to the metrics established by the force field used. The investigation of the mechanism of action of anxiolytic and anticonvulsant pathways using the arjunolic acid compound employed the molecular docking technique, in which the interaction behavior of the ligands when associated with their respective target receptors was evaluated, with their respective 3D coordinates deposited on the RCSB Protein Data Bank virtual platform ([https://www.rcsb.org/] [47]. The crystal structure of carbonic anhydrase II (CAII), linked to anticonvulsant activity, with identification code IDPDB: 3F8E, and titled 'Coumarins are a novel class of suicide carbonic anhydrase inhibitors', this receptor is related to the organism Homo sapiens, obtained through the x‐ray diffraction method (2.0 Å) [38, 48]. For GABA_A_R (anxiolytic), it is pointed out that the 3D infractions of the receptor in question are duly deposited on the platform through the application of the code IDPDB: 6HUP, presented as 'CryoEM structure of human full‐length alpha1beta3gamma2L GABA_A_R in complex with diazepam (Valium), GABA and megabody Mb38', associated with the organisms Homo sapiens and Escherichia coli, and its structural determination has the support of the electron microscopy method (3.58 Å) [49]. In molecular docking simulations, it is necessary to standardize the respective biological receptors studied (CAII and GABA_A_R). Thus, in the first step, the Chimera software [50] is used to remove all non‐protein residues that are inserted in the receptor (ligands and water molecules). With the receptor adjustments, both proteins were added to the AutoDockToolsTM software, in order to add the hydrogens and then add the Gasteiger charges [50]. Subsequently, the axes of the Gridbox (x, y, and z) were adjusted, duly adapted to encompass the entire conformational space of the respective proteins. For CAII, the grid‐box values are delimited equal to 50Åx51Åx56Å (x, y, z) in coordinates x = ‐6.640, y = ‐0.168, and z = 16.240; while for GABA_A_R, the gridbox has dimensions 126Åx100Åx125Å (x,y,z) referring to the coordinates x = 124.280, y = 140.528, and z = 135.020. In the predictions by molecular docking, the AutoDockVinaTM software is applied, in which 50 independent simulations were performed for the ligand, in which each simulation provides 20 distinct possibilities, to compare the bestposes formed regarding the affinity between the arjunolic acid compound associated with both receptors evaluated. The affinity analysis was based on the respective crystallized inhibitors TE1 (CAII inhibitor) and Diazepam (GABA_A_R modulator), with the objective of creating an interaction route to evaluate the interaction profile of the arjunolic acid compound. For the choice of the best pose, the criterion is based on the deviation (RMSD), involving standard values equal to or less than 2.0 Å [51].

In silico analyses were conducted using human carbonic anhydrase II (CA II) and GABA A receptor structures, selected due to the high structural conservation of their binding sites among vertebrates [52]. In addition to demonstrating that these proteins have stable and comparable conformational organization across species, which provides a robust rationale for the utilization of human models in predictive mechanistic investigations. Consequently, in silico modelling facilitates the identification of relevant molecular interactions between ligands and amino acid residues present at the binding site, thereby establishing a rational basis at the atomic level for the pharmacological effects observed in in vivo models [53].

### 4.11 Molecular Dynamics

Molecular dynamics simulations based on Normal Mode Analysis (NMA) were performed using two receptors, GABA_A_ and CAII, in complex with the reference compounds DZP and arjunolic acid (GABA_A_) and TE1 and arjunolic acid (CAII), through the application of the iMODS server (https://imods.iqf.csic.es/). This approach was employed to investigate the structural flexibility and stability of the protein–ligand complexes generated from molecular docking simulations. The complex structures, in PDB format, were submitted to the server for NMA execution, allowing the evaluation of intrinsic conformational motions of the system, thereby providing relevant information on structural stability and functional movements associated with protein–ligand interactions [54]. Within the context of NMA, morphing analysis was used to examine conformational differences between two distinct structures, considering the protein–ligand complex obtained from molecular docking as the initial conformation and the original crystallographic structure as the reference conformation [55].

## Author Contributions

**Yanna Julie da Silva Freitas**: investigation, writing, review, and editing. **Jéssica Bezerra Maciel** and Simone Alves Serafim Rocha: supervision, formal analysis, and software. **Maria Eduarda Uchoa Bezerra**, cléia rocha de Sousa Feitosa, and Andreia Ferreira de Castro Gomes: writing the original draft and reviewing the manuscript. **Emmanuel Silva Marinho** and márcia machado marinho: software and validation. **Jane Eire Silva Alencar de Menezes** and **Hélcio Silva dos Santos**: administration and project writing. **Victor Moreira de Oliveira** and **Otília Deusdênia Loiola Pessoa**: extraction, isolation of samples, and characterization of samples.

## Conflicts of Interest

The authors declare no conflicts of interest.

## Data Availability

The data that support the findings of this study are available from the corresponding author upon reasonable request.
